# Infarction volume modulates and corrects left hemisphere bias in the NIHSS for middle cerebral artery stroke

**DOI:** 10.3389/fnins.2026.1873379

**Published:** 2026-08-12

**Authors:** Dun Pan, Zhengkai Zhao, Qian Liang, Jian Liu, Jin Gao, Hong Liu, Ya Li, Gang Xie

**Affiliations:** Department of Radiology, The Third People’s Hospital of Chengdu, Chengdu, China

**Keywords:** bias, infarction volume, ischemic stroke, middle cerebral artery, NIHSS

## Abstract

**Background and purpose:**

The National Institutes of Health Stroke Scale (NIHSS) exhibits a left hemisphere bias in middle cerebral artery (MCA) stroke. This study aimed to quantify this bias, investigate its modulation by infarction volume, and develop a correction model.

**Methods:**

This retrospective study included 263 first-ever MCA stroke patients, with 173 in the training set and 90 in the test set. Baseline NIHSS scores were compared between left and right hemispheric stroke before and after propensity score matching. Negative binomial regression tested the interaction between infarction side and volume on NIHSS scores in the training set. A correction was then applied to NIHSS scores for left hemisphere strokes. Correction effectiveness was verified by comparing interhemispheric difference in before and after corrected scores, correlation with infarction volume and predictive value for hemorrhagic transformation (HT).

**Results:**

Left hemisphere stroke had higher NIHSS scores than right before and after matching, both in the training and test sets (*p* < 0.05). A significant negative interaction between infarction side and volume (*p* < 0.05) indicated that larger infarction volume attenuates the left hemisphere bias in the NIHSS scores. After correction, no significant interhemispheric difference in NIHSS scores remained (*p* > 0.05). Corrected scores showed stronger correlation with infarction volume (training set: *r* = 0.509 for NIHSS1 and 0.483 for NIHSS2 vs. 0.293 pre-correction; test set: *r* = 0.715 and 0.675 vs. 0.562) and better HT prediction (training set: AUC = 0.688 for NIHSS1 and 0.703 for NIHSS2 vs. 0.653 pre-correction; test set: AUC = 0.747 and 0.757 vs. 0.723) than original NIHSS.

**Conclusion:**

Infarction volume modulates the left hemisphere bias of the NIHSS scores in MCA stroke. The infarction volume-based correction eliminates this bias, strengthens biological validity, and improves the prediction of clinically relevant outcomes like HT.

## Introduction

Acute ischemic stroke (AIS) remains a major global public health challenge due to its high mortality and disability rates (Valery et al., 2021; Valery et al., 2024). The National Institutes of Health Stroke Scale (NIHSS) has become the “gold standard” for assessing stroke severity and prognosis in clinical practice and research, playing a crucial role in treatment decision-making due to its standardization and reliability (Cheng et al., 2025; Anderson et al., 2025; Strambo et al., 2024; Prabhakaran et al., 2026; Berge et al., 2021). However, the NIHSS score has an inherent left hemispheric bias. Numerous studies have confirmed that patients with left hemisphere stroke typically have significantly higher NIHSS scores than those with right hemisphere stroke, particularly in middle cerebral artery (MCA) stroke (Li et al., 2021; Vitti et al., 2022). Consistent with this phenomenon, for the same NIHSS score, infarction volume in the right hemisphere is often larger than in the left (Woo et al., 1999; Fink et al., 2002; Deb-Chatterji et al., 2022). This bias stems from the left hemisphere’s dominance for language in most populations, making the NIHSS more sensitive to language dysfunction caused by left hemisphere damage (Vitti et al., 2022). In contrast, symptoms such as spatial neglect resulting from right hemisphere damage are relatively underrepresented in NIHSS assessment (Silva et al., 2024). This systematic bias may have serious clinical consequences: it could lead to overly aggressive treatment for left hemisphere stroke while causing patients with non-dominant (right) hemisphere stroke to miss appropriate intervention opportunities due to “insufficient scores” (Di Legge et al., 2005; Desai et al., 2018; Almekhlafi et al., 2019). Simultaneously, this bias may compromise the scientific validity and reliability of clinical trial outcomes that employ the NIHSS score as an inclusion criterion (Zhang et al., 2025).

Although hemispheric bias in NIHSS score was well recognized, existing studies have mostly remained at descriptive comparisons, rarely quantifying this bias after rigorously matching key confounding factors (such as infarction volume, age, and sex), and effective correction strategies are lacking (Lyden et al., 2004; Li et al., 2021). Furthermore, an insufficiently explored question is whether the hemispheric bias in NIHSS scores is modulated by infarction volume. Given the ceiling effect of the total NIHSS score, when neurological deficits are extremely severe (massive infarction volume), score differences between hemispheres may be masked (Muir et al., 1996). In other words, hemispheric bias may be more pronounced in small-to-medium infarcts and diminish or even disappear in large infarcts.

Based on the above research gaps, this study aimed to: (1) objectively quantify hemispheric differences in NIHSS scores in patients with unilateral MCA stroke through rigorous matching of confounding factors; (2) further investigate the modulating effect of infarction volume on this bias; and (3) develop a statistical correction model based on infarction volume to eliminate hemispheric bias, thereby enhancing the correlation between NIHSS scores and infarction volume and improving their predictive value for clinical outcomes.

## Materials and methods

### Patients

This retrospective study followed the ethical standards of the Declaration of Helsinki and was approved by the Medical Ethics Committee of The Third People’s Hospital of Chengdu (Approval No. [2025-S-359]), with a waiver of written informed consent.

**Figure 1:**
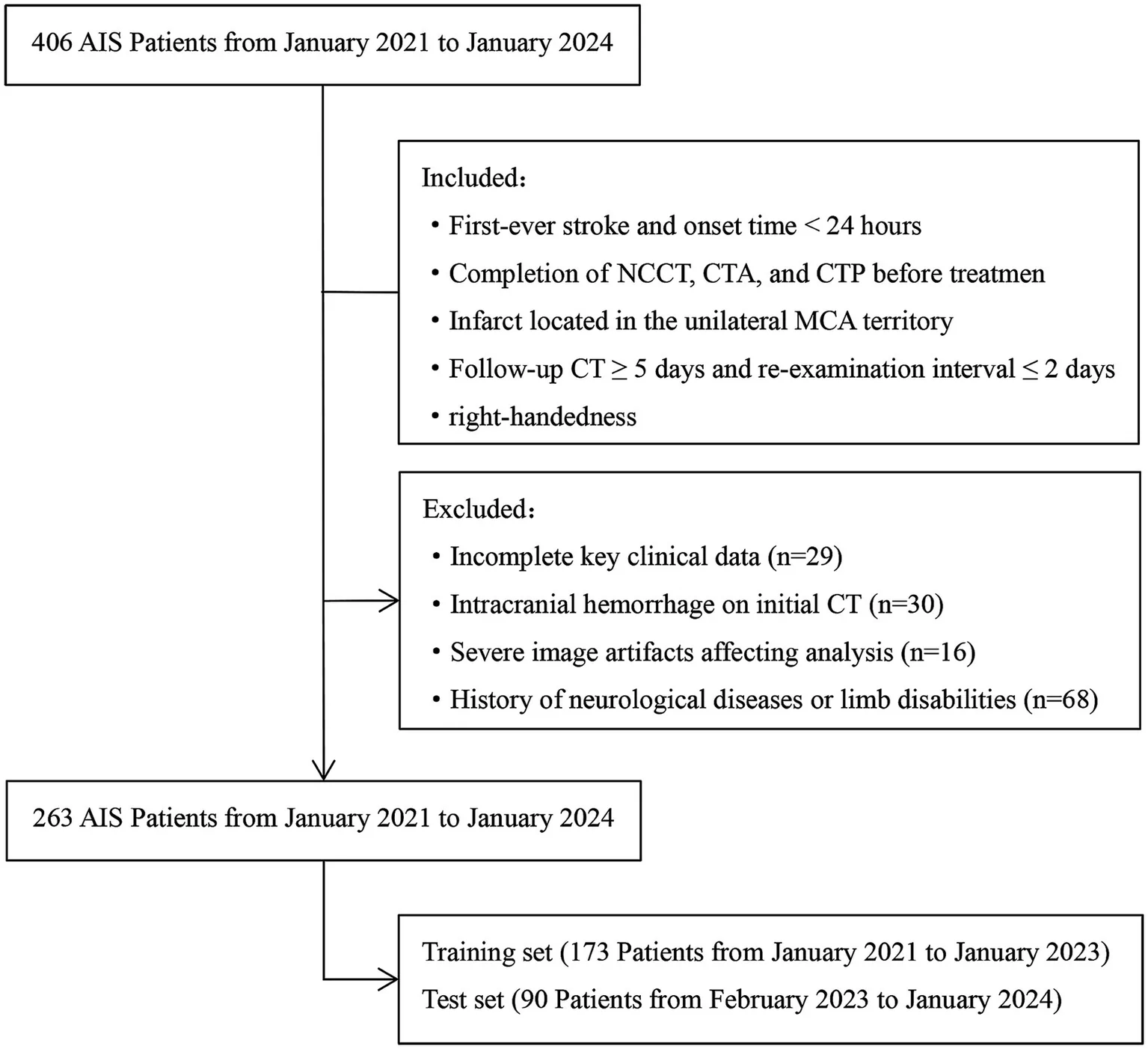
Study flowchart showing patient selection process.

We retrospectively analyzed patients with AIS who received diagnosis and treatment at our center from January 2021 to January 2024. Inclusion criteria were: (1) first-ever stroke, with interval from onset to initial head CT < 24 h; (2) completion of non-contrast CT (NCCT), CT angiography (CTA), and CT perfusion (CTP) in a single session before treatment; (3) infarct located in the unilateral MCA territory, and CTA confirming the culprit vessel as unilateral MCA or its branches; (4) In order to observe the occurrence of hemorrhagic transformation (HT), the initial CT was used as the baseline. Serial follow-up CT was performed for a total duration of at least 5 days, with an interval of no more than 2 days between consecutive scans; and (5) right-handedness. Exclusion criteria were: (1) incomplete key clinical data (including NIHSS scores); (2) intracranial hemorrhage already present on initial CT; (3) severe image artifacts affecting analysis; and (4) history of neurological diseases or limb disabilities. The patient selection process is shown in Figure 1.

### Data collection

Image acquisition was performed using a Philips 256-slice spiral CT (Brilliance iCT). Non-contrast head CT was performed first, followed by CTP and CTA scans after injection of non-ionic iodinated contrast medium (Iomeprol, 400 mgI/mL) and an equal volume of saline via an antecubital vein using a dual-head power injector at a flow rate of 5.0 mL/s. Detailed scanning parameters are provided in Supplementary material 1.

All imaging data were independently analyzed by two radiologists with >5 years of experience in neuroimaging diagnosis. Recorded variables included infarction side, infarction volume, Alberta Stroke Program Early CT Score (ASPECTS), basal ganglia involvement, dense MCA sign, and HT. Using the initial CT as a baseline, patients underwent follow-up CT scans for ≥ 5 days after admission. HT was confirmed if high-density areas (CT attenuation values approximately 60–90 Hu) were observed on CT for two consecutive days or longer. Infarction volume (Defined as cerebral blood flow < 30% of the contralateral normal brain tissue area) was automatically obtained using Shukun artificial intelligence software (Zhou et al., 2023). Consistency assessment between the two readers is shown in Supplementary material 2; discrepancies were resolved through consensus.

Clinical data were extracted from the electronic medical record system, including sex, age, stroke onset time, and baseline NIHSS scores. NIHSS scores were assessed by trained neurologists strictly following standard procedures (Lyden, 2017).

### Analysis of hemispheric bias in NIHSS scores

Patients were divided into two groups based on infarction side (left vs. right) in the training and test sets. To account for confounding factors potentially associated with NIHSS scores, propensity score matching (PSM) was performed in both the training and test sets to match patients from both groups at a 1:1 ratio using a caliper width of 0.01, reducing confounding bias. Covariate balance between groups was assessed after matching. NIHSS score differences between left and right hemisphere stroke patients in the training and test sets were compared before and after matching, and correlations between infarction volume/ASPECTS and NIHSS scores were analyzed in all patients before matching using Spearman correlation coefficients.

### Impact of infarction volume on hemispheric bias of the NIHSS score

To investigate the modulating effect of infarction volume on hemispheric differences in NIHSS scores, we constructed a generalized linear model in the training set using negative binomial regression. The model used the NIHSS score as the dependent variable, and infarction side, infarction volume, and their interaction term as core independent variables, while adjusting for other covariates (e.g., age, sex, stroke onset time) in the training set samples (before matching). The model expression was:

$$\begin{array}{l} \log(\text{NIHSS})=\beta_{0}+\beta_{1}\times I(\text{left})+\beta_{2}\times (\text{Infarction volume})+\\ \beta_{3}\times I(\text{left})\times (\text{Infarction volume})+\Sigma \gamma_{i}\times \text{Covariates} \end{array}$$

A statistically significant interaction term *β*₃ (*p* < 0.05) would indicate that the effect of each unit increase in infarction volume on the NIHSS score differed by hemisphere, i.e., infarction volume modulated hemispheric bias in NIHSS scores. Based on the model, the critical value of infarction volume when the NIHSS scores of the left and right hemispheres were equal was estimated, and subgroup analysis was conducted accordingly to compare the hemispheric differences in NIHSS scores among different infarction volume subgroups.

### NIHSS score correction and validation

Based on the above generalized linear model, the portion of the left hemisphere score exceeding the “expected right hemisphere score for the same infarction volume” was estimated as the “bias value.” For left hemisphere patients with infarct volumes < the critical value, this bias was subtracted from their original NIHSS score to obtain the corrected score. The bias and correction formulas are presented below; detailed derivations are provided in Supplementary material 3.

$$\text{NIHSS}\_\text{bias}=\text{NIHSS}\times [1-\exp(-\beta_{1}-\beta_{3}\times \text{infarction volume})]$$

$$\text{NIHSS}\_\text{corrected}=\text{NIHSS}\times \exp(-\beta_{1}-\beta_{3}\times \text{infarction volume})$$

Correction effectiveness was validated through three aspects: (1) comparing whether statistically significant differences in NIHSS scores between left and right hemispheres remained after correction; (2) comparing Spearman correlation coefficients between NIHSS scores and infarction volume before and after correction; and (3) using receiver operating characteristic (ROC) curves to evaluate the predictive performance of pre- and post-correction NIHSS scores for HT, calculating the area under the curve (AUC) and comparing them using DeLong’s test. Additionally, to verify the directionality of correction, a reverse correction control was performed: artificially adding the same bias value to right hemisphere NIHSS scores and evaluating whether their predictive performance for HT was superior to original scores.

### Statistical analysis

Data analysis was performed using SPSS (version 26.0, RRID:SCR_016479). For continuous data, normally distributed variables were expressed as mean ± standard deviation (SD), while non-normally distributed variables were expressed as median (interquartile range [IQR]). Categorical data were expressed as frequencies and constituent ratios (%). For between-group comparisons of continuous data, the two independent samples *t*-test was used for normally distributed data; otherwise, the Mann–Whitney *U* test was used. For between-group comparisons of categorical data, the chi-square test or Fisher’s exact test was used. Statistical significance was set at two-sided *p* < 0.05.

## Results

### Baseline characteristics of patients

During the study period, a total of 406 patients were initially included, of whom 143 were excluded. The final study population comprised 263 patients, chronologically partitioned into a training set (*n* = 173) and a test set (*n* = 90). Baseline characteristics of all patients are shown in Table 1. There were 156 males and 107 females, with a median age of 74 years and median stroke onset time of 5 h. All patients received standard reperfusion therapy (such as intravenous thrombolysis, mechanical thrombectomy or bridging therapy, etc.) and/or non-reperfusion therapy (such as anticoagulation, antiplatelet therapy, etc.) after admission. HT occurred in 124 patients (47.1%).

**Table 1:** Baseline characteristics of patients before propensity score matching.

Variables	Training set			Test set		
	Left (74)	Right (99)	*p* value	Left (47)	Right (43)	*p* value
Age	73.0 (64.0, 79.0)	73.0 (64.5, 79.5)	0.817	77.0 (68.0, 83.0)	74.0 (63.0, 80.0)	0.130
Infarction volume	104.0 (42.0, 178.6)	69.0 (30.5, 128.5)	0.021	19.6 (4.2, 98.8)	50.0 (8.6, 187.4)	0.132
ASPECTS	4.0 (1.0, 7.0)	5.0 (2.0, 7.0)	0.410	7.0 (5.0, 9.0)	7.0 (3.0, 8.0)	0.461
Stroke onset time	5.0 (2.0, 10.0)	4.0 (3.0, 8.0)	0.646	5.0 (4.0, 6.3)	6.0 (4.0, 10.0)	0.093
Sex			0.786			0.168
Female	29 (39.2%)	42 (42.4%)		22 (46.8%)	14 (32.6%)	
Male	45 (60.8%)	57 (57.6%)		25 (53.2%)	29 (67.4%)	
Basal ganglia area involvement			0.070			0.012
No	27 (36.5%)	51 (51.5%)		23 (48.9%)	10 (23.3%)	
Yes	47 (63.5%)	48 (48.5%)		24 (51.1%)	33 (76.7%)	
Dense MCA Sign			0.893			0.718
No	42 (56.8%)	54 (54.5%)		28 (59.6%)	24 (55.8%)	
Yes	32 (43.2%)	45 (45.5%)		19 (40.4%)	19 (44.2%)	

Data are expressed as median (interquartile range) or frequency (percentage). ASPECTS, Alberta Stroke Program Early CT Score; Dense MCA sign, dense middle cerebral artery sign.

### Analysis of hemispheric bias in the NIHSS score

We performed 1:1 PSM between patients with left and right hemispheric stroke, incorporating infarction volume, ASPECTS, stroke onset time, age, sex, basal ganglia involvement, and dense MCA sign as matching covariates. This yielded 74 matched pairs in the training set and 31 pairs in the test set. As presented in Table 2, all covariates were well balanced between the two hemispheric subgroups in both sets after matching (all *p* > 0.05), which verified the satisfactory performance of the matching procedure.

**Table 2:** Baseline characteristics of patients after propensity score matching.

Variables	Training set			Test set		
	Left (74)	Right (74)	*p* value	Left (31)	Right (31)	*p* value
Age	73.0 (64.0, 79.0)	71.0 (61.0, 80.0)	0.793	73.0 (66.0, 81.0)	75.0 (65.0, 80.0)	0.855
Infarction volume	104.0 (42.0, 178.6)	97.0 (42.0, 145.0)	0.368	34.2 (4.0, 98.8)	50.0 (8.6, 151.4)	0.349
ASPECTS	4.0(1.0, 7.0)	4.0 (2.0, 6.0)	0.714	7.0 (5.0, 9.0)	7.0 (4.0, 8.0)	0.600
Stroke onset time	5.0 (2.0, 10.0)	4.0 (3.0, 9.0)	0.871	5.1 (4.0, 9.5)	6.0 (4.0, 10.0)	0.810
Sex			0.616			0.596
Female	29 (39.2%)	32 (43.2%)		10 (32.3%)	12 (38.7%)	
Male	45 (60.8%)	42 (56.8%)		21 (67.7%)	19 (61.3%)	
Basal ganglia area involvement			0.315			0.282
No	27 (36.5%)	33 (44.6%)		12 (38.7%)	10 (32.3%)	
Yes	47 (63.5%)	41 (55.4%)		19 (61.3%)	21 (67.7%)	
Dense MCA Sign			0.249			0.067
No	42 (56.8%)	35 (47.3%)		19 (61.3%)	18 (58.1%)	
Yes	32 (43.2%)	39 (52.7%)		12 (38.7%)	13 (41.9%)	

Data are expressed as median (interquartile range) or frequency (percentage). ASPECTS, Alberta Stroke Program Early CT Score; Dense MCA sign, dense middle cerebral artery sign.

**Figure 2:**
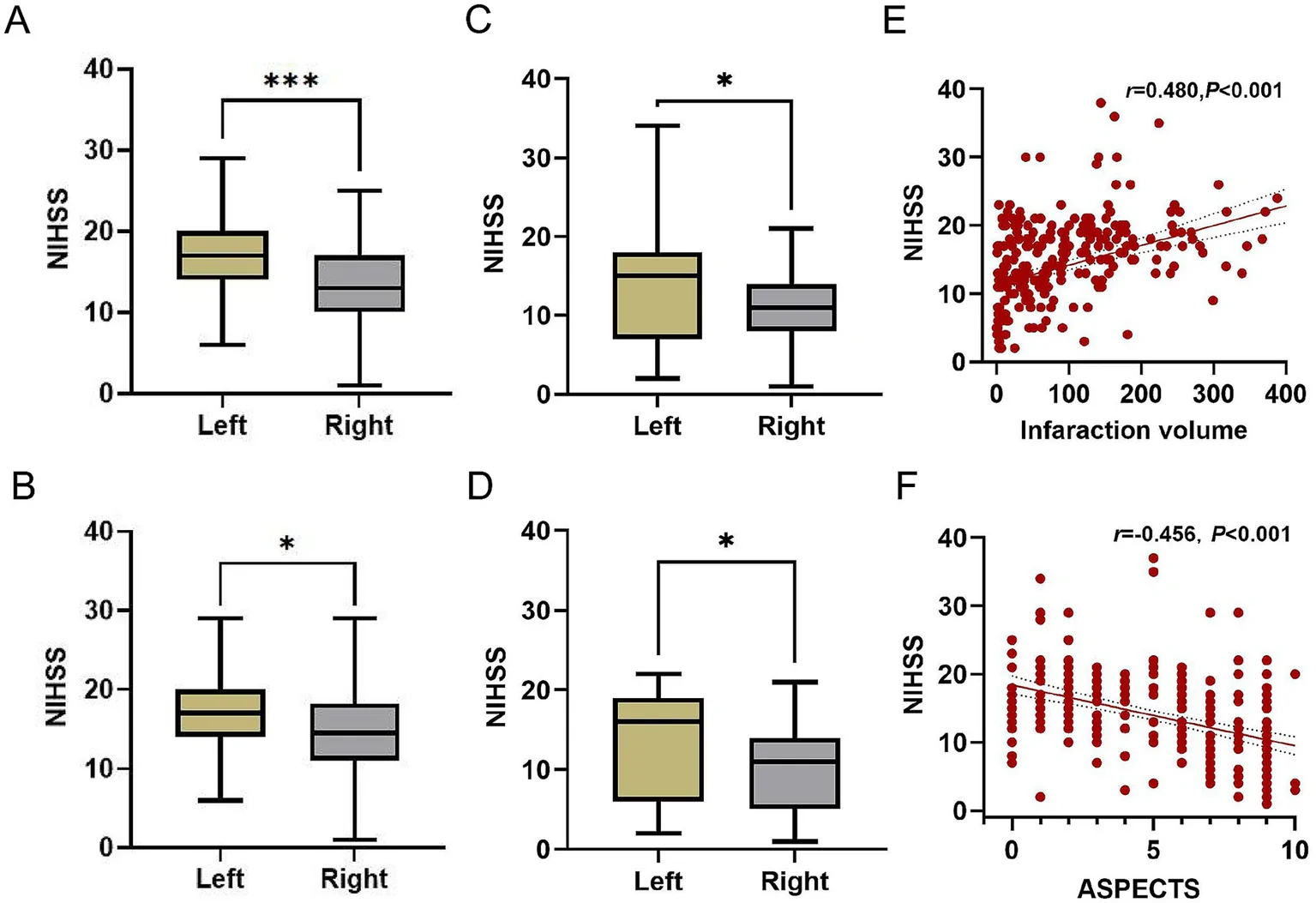
Hemispheric comparison and correlation analysis of NIHSS scores. Comparison of NIHSS scores between patients with left and right hemispheric stroke before **(A)** and after **(B)** matching in the training set, as well as before **(C)** and after **(D)** matching in the test set. **(E,F)** Spearman’s correlation between infarction volume/ASPECTS and NIHSS scores among all patients before matching. *** *p* < 0.001, * *p* < 0.05.

Box plots depict the NIHSS score differences between patients with left and right hemispheric stroke before and after matching (Figures 2A–D). In the training set, left-sided strokes consistently outscored right-sided strokes, both pre-matching [17.0 (14.0, 20.0) vs. 13.0 (10.0, 17.0), *p* < 0.001] and post-matching [17.0 (14.0, 20.0) vs. 14.0 (11.0, 18.0), *p* < 0.05]. Similar findings were obtained in the test set, where the left–right disparity was also significant before [15.0 (7.0, 18.0) vs. 11.0 (8.0, 14.0), *p* < 0.05] and after matching [16.0 (7.0, 18.0) vs. 11.0 (6.0, 13.0), *p* < 0.05]. Before matching, NIHSS scores of all patients were positively correlated with infarction volume (*r* = 0.480, *p* < 0.001) and negatively correlated with ASPECTS (*r* = −0.456, *p* < 0.001) (Figures 2E,F).

### Impact of infarction volume on hemispheric bias of the NIHSS score

Negative binomial regression analysis in Table 3 showed that regardless of whether covariates (e.g., age, sex, stroke onset time) were adjusted, the interaction term between infarction side and infarction volume was statistically significant (interaction term *β*₃: −0.0043 and −0.0015, respectively, *p* < 0.05), indicating that infarction volume exerts a negative modulating effect on left hemisphere bias in the NIHSS score. That is, as infarction volume increased, the difference in NIHSS scores between left and right hemispheres gradually diminished.

**Table 3:** Generalized linear model of the interaction effect between the infarction side and volume.

Models	Intercept (*β*₀)	Infarction side (*β*₁)	Infarction volume (*β*₂)	Side (left): Infarction volume (*β*₃)
Model 1	1.9041***	0.4429***	0.0079***	−0.0043**
Model 2	2.7512***	0.3150***	0.0024***	−0.0015*

* Model 1: Generalized linear model using negative binomial regression with core independent variables including infarction side, infarction volume, and their interaction term. Model 2: Model 1 adjusted for age, sex, and stroke onset time. * *p* < 0.05; ** *p* < 0.01; *** *p* < 0.001.

**Figure 3:**
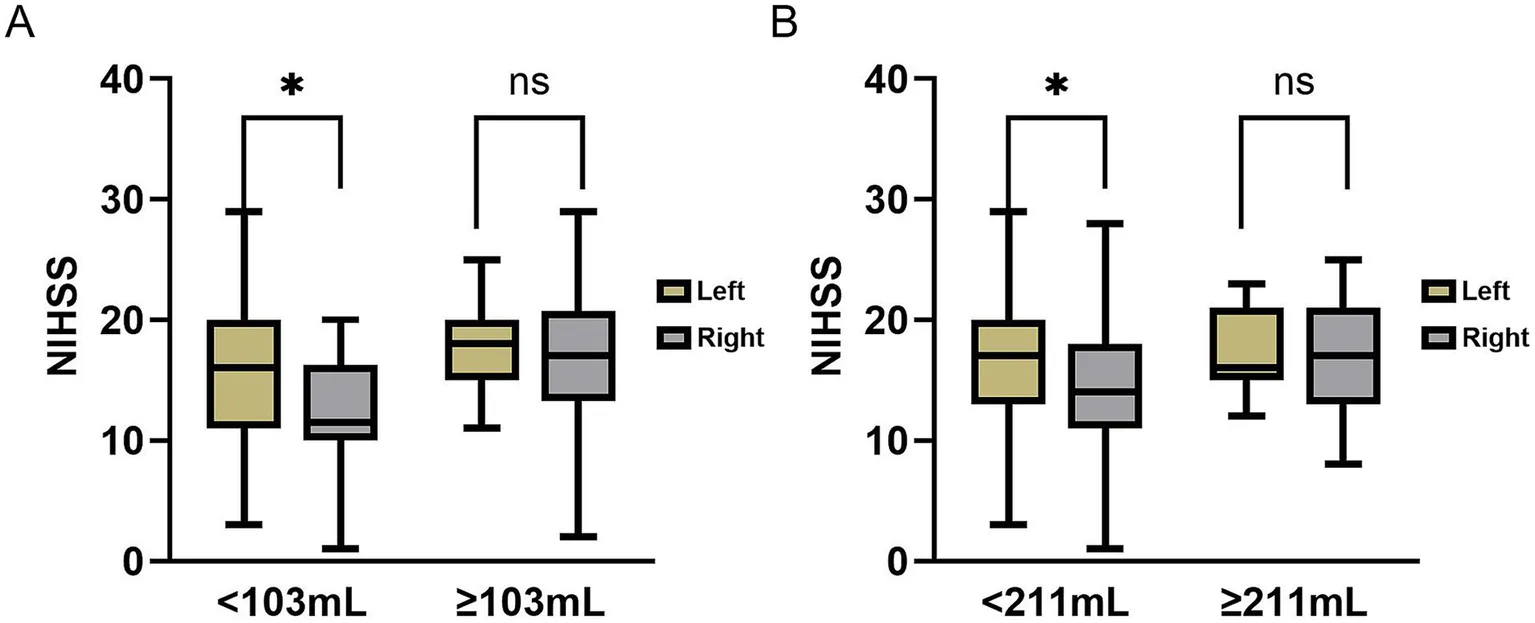
Subgroup analysis of NIHSS scores based on critical infarction volume thresholds. **(A)** Patients stratified by 103 mL threshold (Model 1). **(B)** Patients stratified by 211 mL threshold (Model 2). ns, not significant; * *p* < 0.05.

Based on the unadjusted model (Model 1) and adjusted model (Model 2), the critical infarction volumes at which left and right NIHSS scores were equal were estimated as 103 mL and 211 mL, respectively. Subgroup analysis in the training set showed that in patients below the critical value (<103 mL or < 211 mL), NIHSS scores were significantly higher in left hemisphere stroke than in right hemisphere stroke; in patients above the critical value (≥103 mL or ≥ 211 mL), the difference between sides disappeared (Figure 3).

### NIHSS score correction and validation

**Figure 4:**
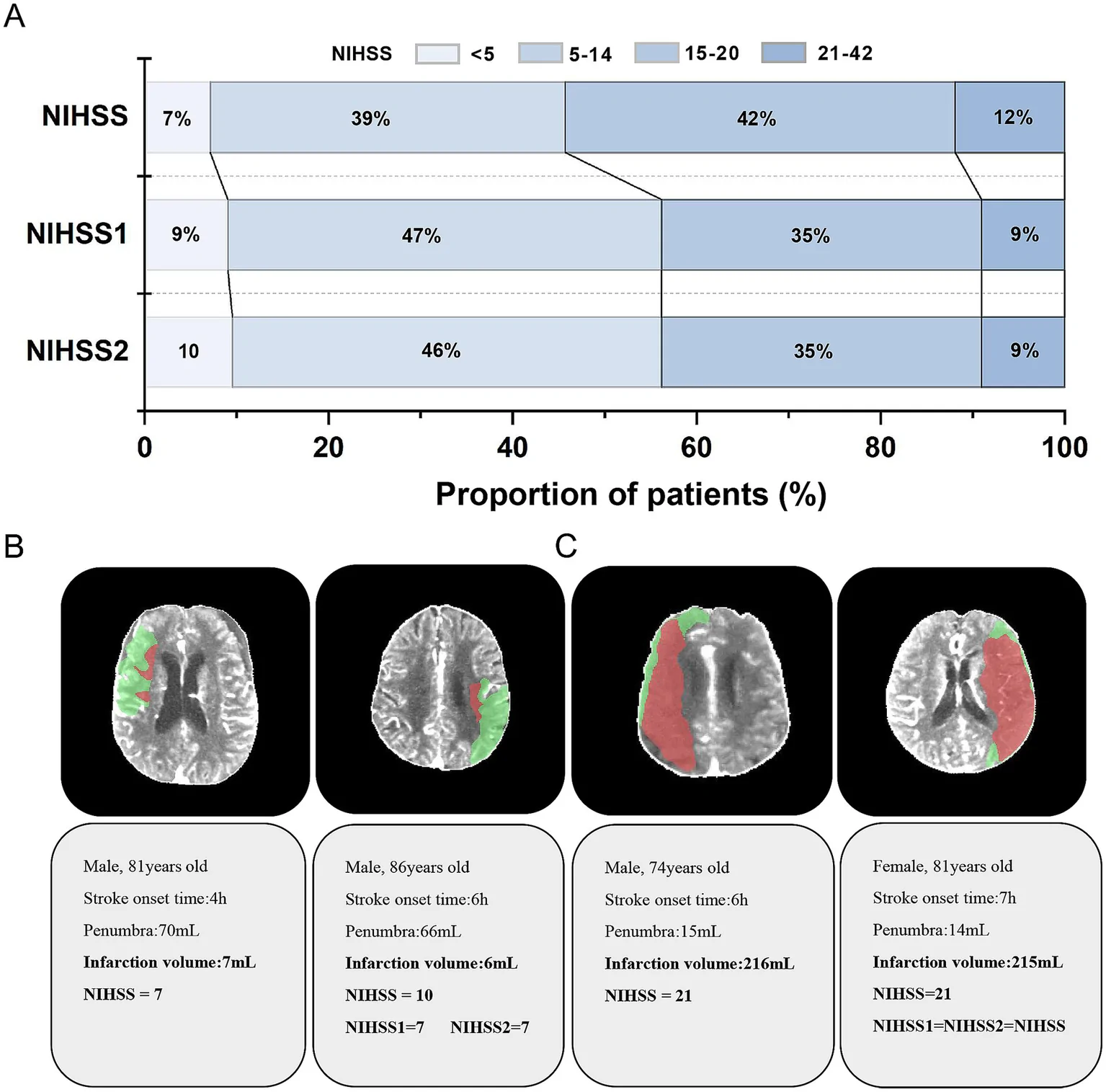
The proportion of NIHSS score categories in left hemisphere stroke patients before and after correction, and examples of NIHSS correction. **(A)** The proportion of patients with NIHSS < 15 increased after correction, while the proportion with NIHSS ≥ 15 decreased. **(B)** For two small infarct AIS patients with comparable infarction volumes, the NIHSS score was higher for the left hemisphere compared to the right hemisphere, but the corrected NIHSS scores were consistent. **(C)** For two large infarct AIS patients with comparable infarction volumes exceeding the critical threshold (>211 mL), the NIHSS scores were similar between the left and right hemispheres.

According to the correction formula, two corrected scores (NIHSS1 and NIHSS2) were calculated using the coefficients from Model 1 and Model 2 in Table 3, respectively. In left hemisphere stroke patients, the proportion of patients with corrected NIHSS < 15 increased, while the proportion with NIHSS ≥ 15 decreased (Figure 4), preliminarily showing that correction reduced the weight of left hemisphere scores.

**Figure 5:**
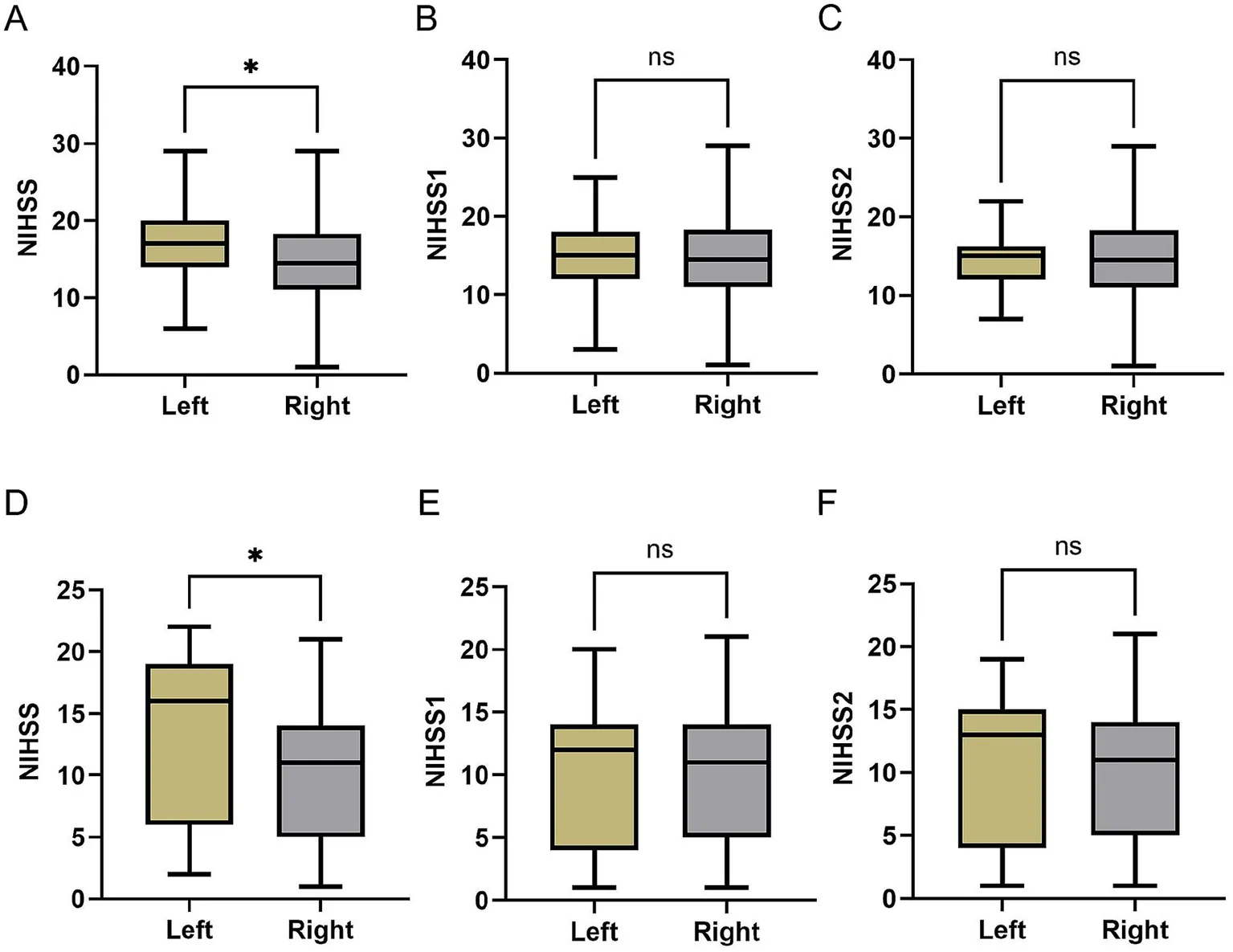
Comparison of NIHSS scores between left and right hemispheric stroke before and after correction. **(A–C)** Comparisons of original NIHSS, corrected NIHSS1, and NIHSS2 scores between left- and right-sided stroke in the training set. **(D–F)** Comparisons of original NIHSS, corrected NIHSS1, and NIHSS2 scores between left- and right-sided stroke in the test set. * *p* < 0.05; ns, not significant.

**Figure 6:**
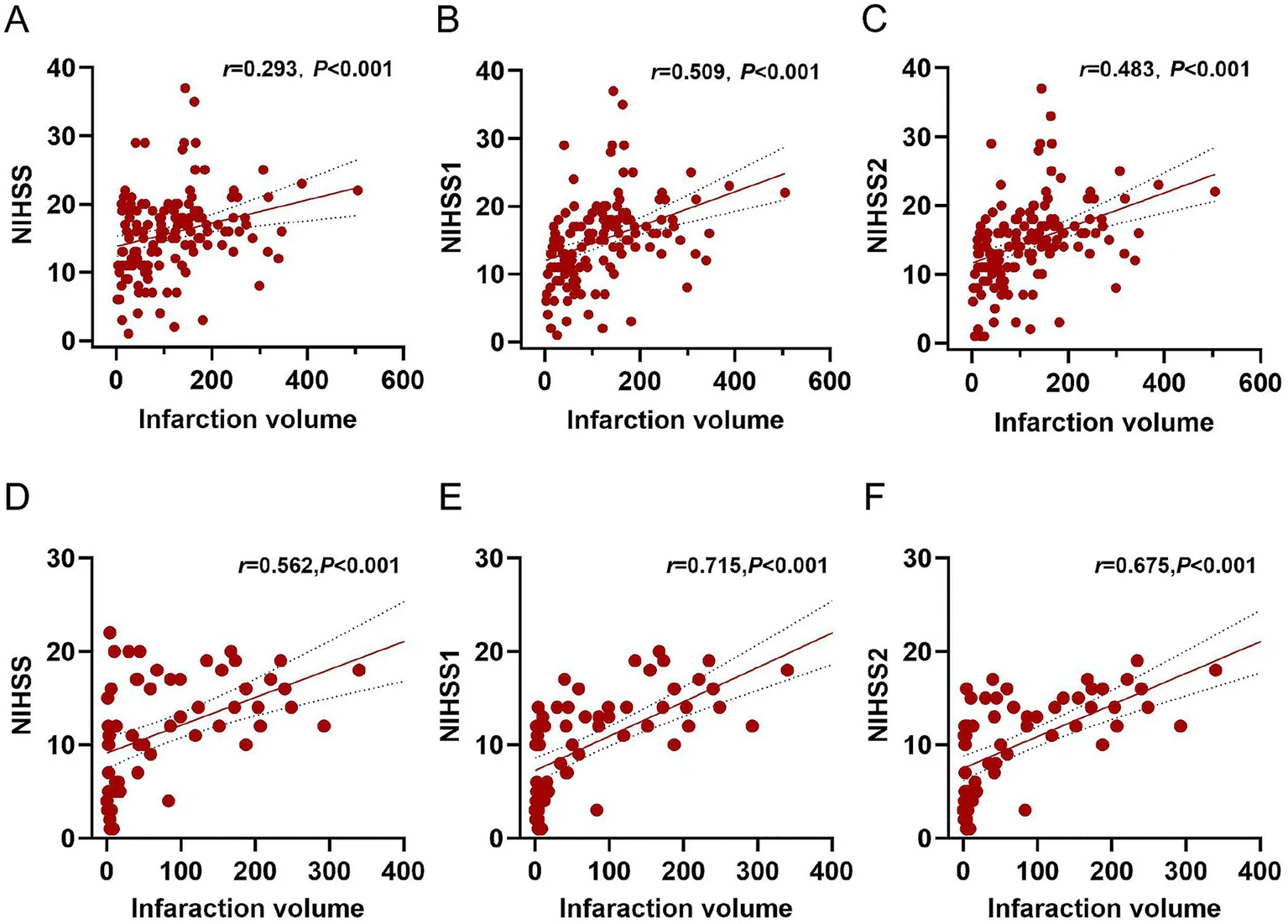
Correlation analysis between infarction volume and NIHSS scores before and after correction. **(A–C)** Spearman correlations of original NIHSS, corrected NIHSS1, and NIHSS2 with infarction volume in the training set. **(D–F)** Spearman correlations of original NIHSS, corrected NIHSS1, and NIHSS2 with infarction volume in the test set.

In validation of hemispheric bias in corrected NIHSS scores in the training and test sets, neither NIHSS1 nor NIHSS2 showed significant differences between left and right hemispheres (*p* > 0.05), indicating effective correction (Figure 5). Spearman correlation analysis revealed substantially strengthened associations with infarction volume after correction. In the training set, correlation coefficients increased from 0.293 (pre-correction) to 0.509 for NIHSS1 and 0.483 for NIHSS2 (both *p* < 0.001; Figures 6A–C). Similarly, in the test set, the coefficients rose from 0.562 to 0.715 and 0.675 for NIHSS1 and NIHSS2, respectively (all *p* < 0.001; Figures 6D–F).

**Figure 7:**
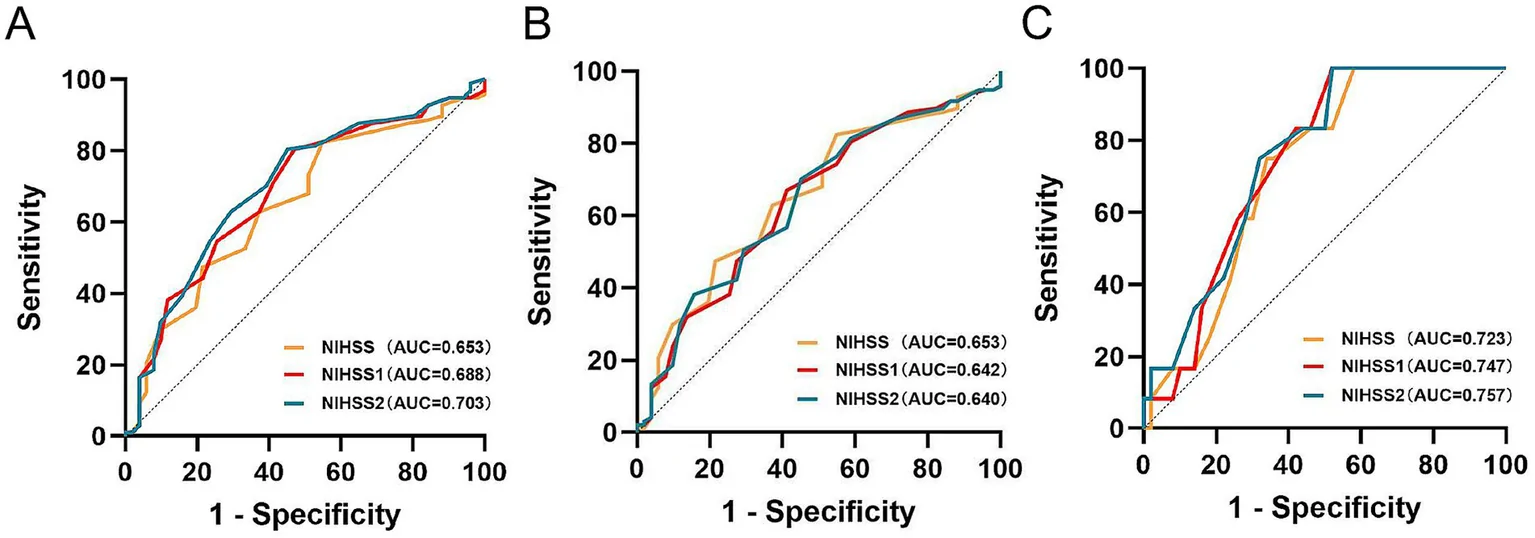
Predictive performance for hemorrhagic transformation (HT). **(A)** ROC curves for HT prediction based on original NIHSS, corrected NIHSS1, and NIHSS2 in the training set. **(B)** ROC curves for HT prediction based on original NIHSS, reverse-corrected NIHSS1, and NIHSS2 in the training set. **(C)** ROC curves for HT prediction based on original NIHSS, corrected NIHSS1, and NIHSS2 in the test set.

In predicting HT, both corrected scores showed superior AUCs to the original NIHSS in the training set (NIHSS1: 0.688 [0.598–0.778]; NIHSS2: 0.703 [0.614–0.791] vs. original: 0.653 [0.561–0.745]), with the NIHSS2 improvement reaching significance (DeLong’s test, *p* < 0.05). Similar trends were observed in the test set (NIHSS1: 0.747 [0.616–0.874]; NIHSS2: 0.757 [0.605–0.866] vs. original: 0.723 [0.586–0.853]), though not statistically significant (Figures 7A,C). Reverse-correction analysis further validated the leftward subtraction approach, as adding the bias to right-sided scores consistently reduced predictive accuracy (Figure 7B).

## Discussion

This study systematically confirmed the presence of significant left hemisphere bias in NIHSS scores among patients with AIS in MCA stroke through rigorous confounding factor matching. It revealed for the first time that this bias is negatively modulated by infarction volume. Building on this finding, we developed and validated a statistical correction model based on infarction volume. This correction model not only effectively eliminated between-hemisphere differences in scores and significantly enhanced the biological correlation between NIHSS scores and infarction volume but also practically improved the predictive ability for HT, an important clinical outcome.

First, this study replicated the previously observed phenomenon of higher NIHSS scores in left hemisphere stroke (Vitti et al., 2022). After rigorously controlling for key confounding factors including infarction volume through PSM, this difference remained stable, strongly suggesting that this bias does not stem from baseline differences in patient populations but is more likely rooted in the structure of the scale itself or the neurological functions it assesses (Silva et al., 2024; Hedna et al., 2013). A notable methodological consideration arises from the substantial difference in infarction volume distribution between the training and test sets. The test set exhibited a markedly smaller median infarction volume compared with the training set. This discrepancy likely reflects temporal improvements in prehospital stroke recognition, emergency medical service activation, and reperfusion therapy efficiency during the study period (2021–2024), rather than selection bias, as the inclusion and exclusion criteria remained strictly consistent across both sets. One might reasonably question whether such a marked volume disparity undermines the validity of the validation exercise. On the contrary, we contend that this difference strengthens, rather than weakens, the evidence for model generalizability. The correction formula—derived from a set with predominantly medium-to-large infarcts—performed effectively and consistently in an independent set with predominantly small-to-medium infarcts. This cross-volume validation constitutes a stringent stress test for our model, as it demands that the correction mechanism operate reliably across a broader spectrum of infarct sizes, including smaller volumes that were underrepresented in the training set.

The core theoretical contribution of this study lies in the first discovery and validation of the negative modulating effect of infarction volume on NIHSS hemispheric bias. Our model further revealed that the interhemispheric NIHSS difference is not static but progressively narrows with increasing infarction volume, vanishing entirely beyond a critical threshold. This pattern is consistent with the prior observation (Fink et al., 2002) that right-hemisphere infarction volume correlated more strongly with NIHSS than did left-hemisphere volume (*r* = 0.62 vs. 0.60). This volume-dependent bias can be understood through a two-stage neuroanatomical framework. First, in small-to-medium infarcts, damage predominantly affects hemisphere-specific functions—left-hemisphere language (contributing up to 7 NIHSS points) versus right-hemisphere spatial neglect—producing asymmetric scores for comparable lesion volumes (Woo et al., 1999; Rangus et al., 2026; Cazzoli et al., 2025). Second, as infarcts enlarge, bilateral core networks subserving consciousness, motor, and sensory functions become progressively disrupted (Sherzad et al., 2025; Frontzkowski et al., 2024). In massive infarcts, these global deficits saturate the scale, generating a plateau effect that masks hemisphere-specific functional differences. Consequently, the left hemisphere exhausts its scoring capacity earlier, losing the ability to distinguish severity beyond a certain infarct size; the right hemisphere—lacking similarly weighted items—can continue accruing points through neglect, motor, and sensory subscales. This differential saturation ultimately causes the interhemispheric NIHSS gap to diminish and eventually disappear at large volumes. Collectively, this finding suggests that future applications of NIHSS for cross-hemisphere comparison of stroke severity should fully consider the modifying effect of infarction volume.

Based on the above interaction effect model, the proposed correction formula has a clear statistical derivation path and biological explanatory power. The significantly enhanced correlation between corrected scores and infarction volume demonstrates that this method effectively removes the interference of hemisphere-specific factors from functional assessment, making the score more purely reflect the “global” neurological deficit load associated with infarction volume. This provides a feasible technical approach for horizontal comparison of severity between patients with different hemisphere strokes. More clinically valuable is the superior predictive performance of corrected scores for HT. HT is one of the most critical complications after reperfusion therapy, and accurate risk assessment directly relates to the safety and effectiveness of treatment decisions (Tian et al., 2022; Pauillac et al., 2025). Due to the inherent hemispheric bias of the original NIHSS score, for any given score, it may systematically overestimate bleeding risk in patients with left hemisphere lesions while underestimating it in those with right hemisphere involvement (Audebert et al., 2011). By eliminating this systematic bias, the corrected scores make the predictive signal for HT clearer and more universal, thus achieving an improved AUC. This result suggests that in future clinical trial design, prognostic model construction, and even intelligent clinical decision support systems, using hemisphere bias-corrected NIHSS scores may enable more accurate risk stratification.

This study has several limitations. First, this was a single-center retrospective study. Although PSM was used to control confounding, potential selection bias and unknown confounding factors cannot be completely eliminated. Conclusions still require external validation in larger, prospective, multi-center cohorts. Second, the correction model did not distinguish between cortical and subcortical infarcts, nor did it refine to NIHSS sub-item scores. The contribution of different infarct locations to different NIHSS items varies significantly (Rajashekar et al., 2022; Garrard et al., 2025). Future studies could combine voxel-based symptom mapping and sub-item analysis to achieve “location-specific” ultra-precise correction. Third, the critical infarction volumes derived from the model differed considerably under different covariate adjustments (103 mL vs. 211 mL), suggesting that this threshold is model-dependent. This threshold only reflects characteristics of the current study population and is not yet suitable for direct clinical decision-making; a stable and reliable cutoff value requires further investigation. Fourth, this study only used HT as an outcome validation indicator; the predictive performance of corrected scores for long-term functional outcomes (such as 3-month modified Rankin Scale scores) remains to be evaluated.

In conclusion, this study goes beyond merely describing hemispheric differences in NIHSS scores, revealing for the first time its dynamic interaction mechanism with infarction volume and successfully constructing an operational statistical correction framework. Application of this correction method can make NIHSS scores more objectively reflect stroke severity across both hemispheres and effectively improve predictive ability for key complications, providing new ideas and methods for precise stratification and individualized decision-making in stroke clinical research.

## Data Availability

The original contributions presented in the study are included in the article/Supplementary material, further inquiries can be directed to the corresponding authors.
