# Supplementary material for: Infarction volume modulates and corrects left hemisphere bias in the NIHSS for middle cerebral artery stroke
Source: Front Neurosci. 2026 Aug 12;20:1873379. doi: 10.3389/fnins.2026.1873379 (PMC13506817; doi:10.3389/fnins.2026.1873379)
Supplement: Supplementary file 1 [file Table_1.DOCX]

Table **1** The CT scanning parameters of this study

| **Parameters** | **NCCT** | **CTA** | **CTP** |
| --- | --- | --- | --- |
| Tube voltage, kV | 120 | 100 | 80 |
| Tube current, mA | 220 | 220 | 100 |
| Slice thickness, mm | 5 | 0.625 | 5 |
| Slice interval, mm | 5 | 0.625 | 5 |
| Collimation width | 128 × 0.625 | 128 × 0.625 | 128 × 0.625 |
| Matrix | 512 × 512 | 512 × 512 | 512 × 512 |

*NCCT, non-contrast CT; CTA, CT angiography; CTP, CT perfusion*
