# Supplementary material for: Infarction volume modulates and corrects left hemisphere bias in the NIHSS for middle cerebral artery stroke
Source: Front Neurosci. 2026 Aug 12;20:1873379. doi: 10.3389/fnins.2026.1873379 (PMC13506817; doi:10.3389/fnins.2026.1873379)
Supplement: Supplementary file 2 [file Table_2.DOCX]

Table **2** Consistency analysis of evaluations by two radiologists

| **Variable** | **Kappa of** **ICC valve** | ***P* value** |
| --- | --- | --- |
| Stroke side | 1.000 | < 0.001 |
| ASPECTS | 0.761 | < 0.001 |
| Basal ganglia involvement | 0.848 | < 0.001 |
| Dense MCA Sign | 0.883 | < 0.001 |

*ASPECTS, Alberta Stroke Program Early CT score; Dense MCA sign, Dense middle cerebral artery sign; ICC, Intraclass correlation coefficient*
