# Supplementary material for: Infarction volume modulates and corrects left hemisphere bias in the NIHSS for middle cerebral artery stroke
Source: Front Neurosci. 2026 Aug 12;20:1873379. doi: 10.3389/fnins.2026.1873379 (PMC13506817; doi:10.3389/fnins.2026.1873379)
Supplement: Supplementary file 3 [file Table_3.DOCX]

**Derivation of the NIHSS Correction Formula:**

Negative binomial regression model (log scale) expression:

*log(NIHSS) = β₀ + β₁×I(Left) + β₂×Infarction volume + β₃×I(Left)×Infarction volume+ Σγᵢ×Covariates*

For left hemisphere stroke:

*log(NIHSS_-left_) = β₀ + β₁ + β₂×Infarction volume + β₃×Infarction volume + Σγᵢ×Covariate*

For right hemisphere stroke:

*log(NIHSS_-right_) = β₀ + β₂×Infarction volume + Σγᵢ×Covariates*

*Bias term = log(NIHSS_-left_) - log(NIHSS_-right_) = β₁ + β₃×Infarction volume*

NIHSS correction:

*log(NIHSS_corrected) = log(NIHSS_-left_) - (β₁ + β₃×Infarction volume)*

Taking the exponential of both sides:

*NIHSS_corrected = exp[log(NIHSS_-left_) - (β₁ + β₃×Infarction volume)]*

*= exp[log(NIHSS_-left_)] × exp[-(β₁ + β₃×Infarction volume)]*

*= NIHSS × exp(-β₁ - β₃×Infarction volume)*

**Derivation of the Bias (Value) Formula:**

*Bias = NIHSS - NIHSS_corrected*

*= NIHSS - NIHSS × exp[-(β₁ + β₃ × Volume)]*

*= NIHSS × [1 - exp(-β₁ - β₃ × Volume)]*
